# A method for determination of muscle fiber diameter using single fiber potential (SFP) analysis

**DOI:** 10.1007/s11517-012-0965-x

**Published:** 2012-10-05

**Authors:** Ewa Zalewska, Sanjeev D. Nandedkar, Irena Hausmanowa-Petrusewicz

**Affiliations:** 1 Nałęcz Institute of Biocybernetics and Biomedical Engineering, Polish Academy of Sciences, Ks. Trojdena 4 Str., 02-109 Warsaw, Poland; 2 Natus Medical Incorporated, 1850 Deming Way, Middleton, WI 53562 USA; 3 Neuromuscular Unit, Mossakowski Medical Research Center, Polish Academy of Sciences, Pawinskiego 5, 02-106 Warsaw, Poland

**Keywords:** Electromyography, Single fiber potential, Negative peak duration, Computer simulation, SFEMG

## Abstract

We have used computer simulation to study the relationship between the muscle fiber diameter and parameters: peak-to-peak amplitude and duration of the negative peak of the muscle fiber action potential. We found that the negative peak duration is useful in the determination of fiber diameter via the diameter dependence of conduction velocity. We have shown a direct link between the underlying physiology and the measurements characterizing single fiber potential. Using data from simulations, a graphical tool and an analytical method to estimate the muscle fiber diameter from the recorded action potential has been developed. The ability to quantify the fiber diameter can add significantly to the single fiber electromyography examination. It may help study of muscle fiber diameter variability and thus compliment the muscle biopsy studies.

## Introduction

Single fiber EMG (SFEMG) is a powerful technique to study the pathophysiology of the motor unit. Two types of measurements are made using this technique: fiber density (FD) and jitter. The FD is useful to study the grouping of muscle fibers of a motor unit within its territory. In neuropathy, FD increases due to reinnervation. The “jitter” measurements are used to assess the efficacy of the neuromuscular transmission [9].

In myopathy, the FD may be increased slightly due to fiber splitting, regeneration of muscle fibers, etc. The jitter is usually normal. In this manner, SFEMG is not particularly useful in the diagnosis of myopathy. It is often used to rule out other diseases such as a neuropathy or a neuromuscular junction disease [9]. A primary change in myopathy is the increased variability of the muscle fiber diameter. This is assessed quantitatively using muscle biopsy studies [2]. There is no electrophysiologic technique to assess this characteristic of the motor unit.

The abnormalities of muscle fiber diameter are observed indirectly on electrodiagnostic studies. On routine needle EMG examination, increased variability gives motor unit potentials with polyphasic waveforms. Atrophy can give low amplitude (in what follows by amplitude we mean the peak-to-peak value of potential change, counted in mV) potentials [1, 8, 9].

The variability of muscle fiber diameter may be studied indirectly by investigating the muscle fiber conduction velocity. The muscle fibers are stimulated directly using an intramuscular needle, and their action potentials are recorded at a few millimeter distance. The latency of the potential is used to compute the velocity [10].

In principle, the shape of the single muscle fiber action potential also contains information about the muscle fiber diameter. In the so-called line source mode, the extracellular muscle fiber action potential *V*(*t*) is computed as [3]:

1 $$ V\left( t \right) = \mathop \int \nolimits \left ( {\varphi \left( {t - \tau } \right)i\left( \tau \right)d\tau}\right) $$

where *i*(*t*) is the transmembrane current and φ is a weight function. The transmembrane current is proportional to the square of the fiber diameter [3]. Thus, larger fibers will produce a larger amplitude potential. The amplitude is also affected by the distance of the fiber from the electrode [4]. Higher the distance, lower is the amplitude. Hence, amplitude alone cannot be used as a marker of the fiber diameter.

The weight function is the potential recorded by the electrode as a unit current source propagates from the endplate to the tendon. The waveform of this function is thus dependent on the propagation velocity of muscle fiber, and hence the fiber diameter. The change in weight function waveform with radial distance and with fiber diameter has not been investigated systematically.

In this study, we have used computer simulation to study the relationship between the muscle fiber diameter and parameters of potential: peak-to-peak amplitude and duration of the negative peak of SFP. These relationships are used to develop a graphical and an analytical tool to estimate the muscle fiber diameter from the recorded action potential. Although our goal is similar, our method of analysis is quite different from that used by Rodriguez et al. [6, 7]. They used a computer model to recursively obtain the best match for measured waveform. This is not always possible in clinical environment. We believe that the graphical method described in this study offers more simplicity and gives a better understanding of the action potential waveform.

## Methods

The line source model described by Nandedkar and Stålberg [3] was used for simulating single muscle fiber action potentials. This model has been tested in simulations of normal and abnormal EMG signals, and shows a good concordance with experimental and clinical recordings [4, 5].

The single fiber potential *V* recorded at the electrode at time *t*, coming from a single fiber, is given by a convolution of a weight function φ(*t*) and current *i*(*t*) given by (1). The effect on the potential *V* of the distance of the electrode from the fiber is described by the weight function, which is defined as follows [3]:

2 $$ \varphi \left( {r,z} \right) = \frac{1}{{4\pi \sigma_{r} }}\frac{1}{{\sqrt {Kr^{2} + z^{2} } }} $$

where σ_*r*_ is the radial conductance and *K* depends on the ratio of axial to radial conductance, *r* and *z* are the radial and axial distance of electrode from the current source. The axial distance *z* is related to time *t* by the speed of propagation of the potential along the fiber: *z* = *vt*, where the velocity of propagation *v* is linearly dependent on fiber diameter.

The action potentials were simulated for various combinations of muscle fiber diameter (25–90 μm) and radial distance (50–500 μm). The range of fiber diameters corresponds to the data from muscle biopsy [2]. In normal muscle the mean value is 50 ± 5 μm and in various neuromuscular disorders it is in range from 25 to 90 μm. The range of radial distances was determined so that the largest fibers would not be closer to the electrode than their radius (50 μm). The electrode records mainly from muscle fibers that are within 400 μm of the recording surface [4]. Hence the maximum radial distance was limited to 500 μm. The velocity of propagation of the current is determined by the diameter of the fiber according to the following formula [3]: $v = 2.2 + 0.05(d - 25)$, where the fiber diameter *d* is in μm and the propagation velocity is in ms^−1^.

For the simulation we have used our own software which is based on the formulation of the simulation method presented by Nandedkar and Stålberg [3]. In this method the potential is a result of the convolution of the weight function and source current given by (1) with the source current described by the formula derived by Nandedkar and Stålberg [3]. Using our software we are able to model the potential from one or more fibers and determine its parameters. In the modeling we have assumed the same parameters for conductance as well as for the source current model as given in [3]. The model has been used by us to examine the properties of SFP in various neuromuscular disorders (Nandedkar et al. [5]).

**Fig. 1:**
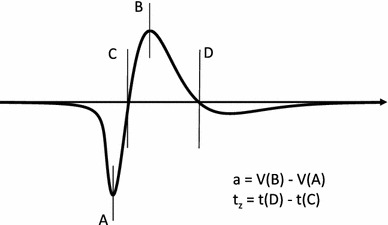
Definitions of the SFP parameters. The amplitude was measured from the maximum positive (*a*) to maximum negative peak (*b*). The time difference between zero-crossings (*c*) and (*d*) is the duration of the negative peak $t_{z}$

The amplitude was measured from maximum positive to maximum negative peaks (Fig. 1). The zero-crossing following the initial downward and subsequent upward peaks were identified. The time difference between them is the duration of the negative peak (Fig. 1)—henceforth it will be denoted by *t*_*z*_.

The change in the amplitude and duration with radial distance and muscle fiber diameter was investigated. In the experimental recordings, the peak duration and amplitude are measured while the muscle fiber diameter and radial distance are the “unknown” variables. Hence we may write:

3 $$ d = d(a,\;t_{z} ) $$

and

4 $$ r = r\left( {a,\;t_{z} } \right) $$

By fixing *t*_*z*_, we obtain parametric curve with coordinates:

5 $$ (d,r)|_{{t_{z} }} = (d(a), r(a)) $$

where the symbol $|_{{t_{z} }}$means that it is a parametric dependence of *d* and *r* on *a* for a fixed *t*_*z*_. Similarly by fixing *a*, we may obtain parametric dependence of *r* and *d* on *t*_*z*_:

6 $$ (d,r)|_{a} = (d(t_{z} ),r(t_{z} )) $$

We have approximated the dependencies between *d*, *r*, *a*, and *t*_*z*_ as follows: the negative peak duration was approximated by the following two bi-quadratic polynomials:

7 $$ t_{z} = F_{1} \left( {\log_{10} \left( a \right), d} \right) $$

and

8 $$ t_{z} = F_{2} \left( {\log_{10} \left( a \right), r} \right) $$

where both $F_{i = 1,2}$ are expressed as:

9 $$ F_{i} \left( {x,y} \right) = e_{i,1} + y\left( {e_{i,2} + ye_{i,3} } \right) $$

where

10 $$ e_{i,j} = c_{i,j,1} + x(c_{i,j,2} + xc_{i,j,3} ) $$

In our approach, the $F_{i}$ are quadratic functions of $y$ which is either fiber diameter $d$ for $i = 1$, or fiber to electrode distance $r$ for $i = 2$. The coefficients of these quadratic polynomials are by themselves given by quadratic Eq. (10) with $x$ equal to the ${ \log }_{10} (a)$. The coefficients $c_{i,j,k}$ are determined by fitting Eqs. (7) and (8) to results of simulation calculations.

We have found experimentally that the form of dependence given by Eqs. (7) and (8) is better suited to least squares bi-quadratic approximation than Eqs. (3) and (4).

In order to calculate the diameter, $x = { \log }_{10} (a)$ has to be calculated from the amplitude of the potential and then used in Eq. (10) to calculate the coefficients $e_{1,1} \ldots e_{1,3}$. Using these coefficients the quadratic Eq. (9) may be solved for $y$ i.e., for the diameter. Since Eq. (9) is quadratic in $y$ it may be solved directly. Similarly by calculating coefficients $e_{2,1} \ldots e_{2,3}$ Eq. (9) may be solved to obtain radius. The same Eq. (9) is used to derive both fiber diameter and radial distance. The quantity that is determined depends on whether one uses the set of coefficients $c_{1,j,k}$—for the determination of $d$, or the coefficients given in the second column in Table 1—for the determination of $r$ from a given $a$ and $t_{z}$.

**Table 1:** The values of the coefficients $c_{1,j,k}$ and $c_{2,j,k}$ used in Eqs. (9) and (10)

$j,k$	$c_{1,j,k}$	$c_{2,j,k}$
1, 1	9.5100E−01	8.3844E−01
1, 2	−2.2321E−01	−5.1557E−01
1, 3	2.0358E−01	8.8151E−02
2, 1	−7.3845E−03	−2.4920E−03
2, 2	3.4212E−04	−1.9468E−04
2, 3	−1.6564E−03	3.2125E−04
3, 1	2.5574E−05	3.4911E−06
3, 2	−3.3074E−06	1.9169E−06
3, 3	6.4493E−06	3.2255E−07

## Results

**Fig. 2:**
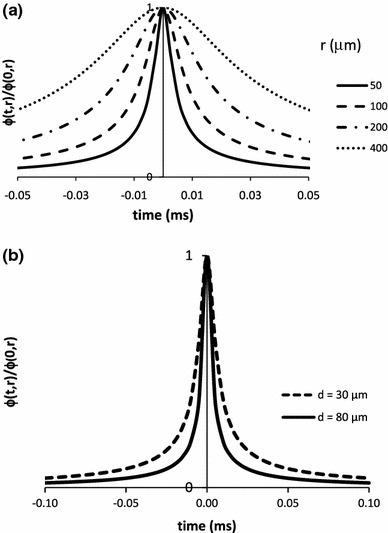
**a** The dependence of the normalized weighting function $\varphi (r,z)$ on axial distance $z$ for several values of the distance $r$ of needle from the fiber. The weighting function has been normalized by its value at $z = 0$. **b** Normalized weight function for muscle fibers of diameters: 30 and 80 μm, located at a distance of 50 μm from electrode. The width of the weight function varies with the diameter of fiber

**Fig. 3:**
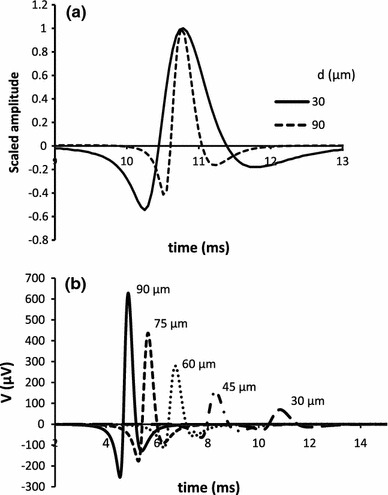
**a** SFP for fiber of diameter 30 and 90 μm located at a distance of 100 μm from the electrode. Both potentials have been scaled so that the maximum = 1. Potential due to larger fiber has been shifted in time (by +5.9 ms) so that the maxima coincide. The increase of the width of potential with the decrease of diameter is clearly seen. **b** SFPs for fibers with diameter of 30, 45, 60, 75, and 90 μm located at a distance of 200 μm from electrode. It is seen how the amplitude of the SFP increases with the increase of fiber diameter and at the same time the potentials from larger fibers arrive earlier at the electrode

**Fig. 4:**
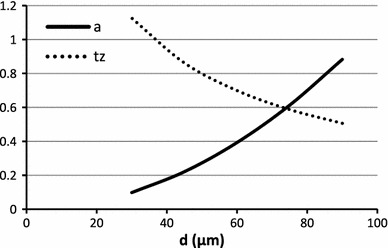
The negative peak duration ($t_{z}$) *dotted line* decreases with the increase in fiber diameter (at fixed fiber to electrode distance). The *solid line* is the dependence of amplitude on fiber diameter. With the increase of diameter the amplitude increases. The amplitude is measured in mV, the negative peak duration is measured in ms

**Fig. 5:**
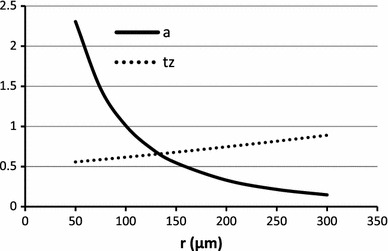
The dependence of $t_{z}$ (*dotted line*) and amplitude (*solid line*) on the electrode distance from the fiber for a fiber of diameter of 55 μm and electrode located at distances from 50 to 300 μm. The amplitude is measured in mV, the negative peak duration is measured in ms. The amplitude decreases with distance and the negative peak duration increases

**Fig. 6:**
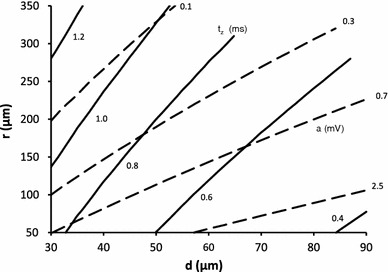
The dependence of fiber diameter (*d*) on fiber to electrode distance (*r*) for a fixed amplitude (*a*) of the potential or a fixed duration $(t_{z} )$. *Solid curves* represent constant duration and *dashed curves* represent constant amplitude. The values of amplitude are given along the *top* and *right margins* of the graph. The values of constant duration are given in the *lower part* of the graph

The results of simulation studies are shown in Figs. 2, 3, 4, 5, and 6. The dependence of the weight function on parameters is shown in Fig. 2. The weight function is broader when the radial distance between the muscle fiber and the recording electrode is increased (Fig. 2a). For the same distance, the smaller fibers have a broader weight function due to slower conduction velocity (Fig. 2b). The simulated waveforms also show differences in the amplitude and durations. When their negative peaks are aligned and their amplitudes are normalized, larger fibers have a shorter negative peak duration (Fig. 3a). With identical radial distance, larger fibers have higher amplitude and shorter duration (Figs. 3b, 4). The amplitude changes much more with radial distance than does the negative peak duration (Fig. 5).

The amplitude and negative peak duration dependencies are shown on a single graph (Fig. 6). The solid curves represent the dependency of $(d,r)|_{{t_{z} }}$ for a range of $t_{z}$ values (0.4–1.4 ms) and dashed curves represent the dependency of $\left. {(d,r)} \right|_{a}$ for a range of amplitude values (50 μV–5 mV). A plot of these dependencies (Fig. 6) allows one to estimate the fiber diameter and radial distance knowing the amplitude and negative peak duration.

For example, if a potential with amplitude of 0.7 mV and a negative peak duration of 0.6 ms is recorded, we could determine the distance and diameter from the intersection of the curve for constant amplitude = 0.7 mV (solid line) and constant duration = 0.6 ms (dashed line). The point of intersection gives the fiber diameter (roughly 65 μm) and the radial distance (150 μm).

While in principle the graphical tool, provided that the curves for constant amplitude or negative peak duration are dense enough, may be used to determine fiber diameter and distance from needle we have approximated the data with analytical formulae which makes them more suitable for use. The graph, however, may be used to verify that the curves are smooth, nearly linear and that the two variables $a$ and $t_{z}$ give rise to two families of curves that are well separated (Fig. 6).

The graph may be readily used to compare the diameters for two or more SFPs. If two SFPs have the same amplitude, so on the graph we are moving along one of the constant amplitude curves, then the one that has shorter negative peak duration is due to larger fiber. This is because the negative peak duration depends on conduction velocity which in turn is proportional to fiber diameter. Hence the larger the fiber, the shorter the negative peak duration.

In order to explain the dependence of fiber diameter on amplitude for a constant negative peak duration one has to note that the amplitude depends primarily on two factors—the distance from the electrode and fiber diameter (see Fig. 3b). For a constant $t_{z}$, the amplitude changes mainly due to the change in fiber to electrode distance. The increase of radius, with the decreasing amplitude, leads to the broadening of the weight function. In order to keep the $t_{z}$ constant the fiber diameter has to increase with the increase of radius. Hence for two SFPs of the same negative peak duration the one for which the amplitude is smaller will be due to a larger fiber.

In order to calculate the values of the coefficients $c_{1,j,k}$ and $c_{2,j,k}$ we have simulated several hundreds of SFP with fiber diameter ranging from 25 to 90 μm and radial distance ranging from 50 to 500 μm calculating for each SFP its peak-to-peak amplitude and negative peak duration. These data were then approximated using formulae (9) and (10) and the coefficients have been determined by standard linear least squares method. These coefficients are given in Table 1. The mean error of the determination of $t_{z}$ from Eq. (7) is $5 \times 10^{ - 3}$, and for Eq. (8) it is $3 \times 10^{ - 3}$ therefore we have rounded the coefficients to 4 significant places.

Using this method to approximate the data it is found that the root mean square error of the determination of diameter is 2 μm and for radial distance it is 6 μm. The maximum error for $d$ is 8 μm, and for $r$ it is 50 μm. The maximum errors occur at the ends of interpolation regions for $r\;>310$ μm, $d<\;35$ μm, or $d\;>125$ μm. Hence the approximations given by Eqs. (7) and (8) enable to determine $d$ and $r$ from $a$ and $t_{z}$ with satisfactory accuracy.

For example, using these equations for a simulated SFP with fiber diameter $$ d = 55$$ μm, fiber to electrode distance $$ r = 80$$ μm for which $$ a = 1.357\;{\text{mV}} $$, hence $$ { \log }_{10} \left( a \right) = 0.13258 $$ and $$ t_{z} = 0.593 $$ it is obtained that $$ d = 55.7$$ μm and $$ r = 80.9$$ μm. In this case the difference between the parameters used to model the SFP and their values obtained from calculation are less than 1 μm.

In order to estimate the effect of measurement errors of $a$ and $t_{z}$ on the result we may assume a measurement error of $\pm 20$ μV for amplitude. For $t_{z}$ we assume an accuracy of $\pm 0.04\;{\text{ms}}$ which corresponds to sampling frequency of 25 kHz. It is found that with the assumed value for the error in $a$ the change in the derived diameter is less than 0.3 μm. By changing $t_{z}$ by the assumed value the $d$ changes by 8 μm. It can be easily verified using the coefficients of the fit that the diameter is more sensitive to measurement errors in $t_{z}$ than to errors in $a$. Therefore, to be able to determine fiber diameter and fiber to electrode distance a good quality of the SFP is required with sufficient resolution in time. It is also for this reason that the graph tool can be only used for approximate or qualitative estimates as it would have to contain tens of constant $t_{z}$ curves to provide comparable accuracy to the presented formulae.

## Discussion

From the simulation studies it has been found that the negative peak duration of SFP is a quantity which is useful in the determination of fiber properties and in particular of fiber diameter. Rodriguez and co-workers [6, 7] have shown that this quantity is related to the width of the source current generating the potential. We have found that $t_{z}$ is related to fiber diameter. This relation is easily understandable in view of the above findings because the duration is proportional to the ratio of the width (~1.5 mm) of the negative peak of the second derivative of intracellular potential [3] and source propagation velocity. The velocity of propagation is dependent on fiber diameter; therefore the larger the diameter, the less time it will take for the feature to pass the electrode, hence the negative peak duration will be shorter. Thus, it turns that $t_{z}$ is one of the basic quantities characterizing the SFP and it gives a direct link to the underlying physiology.

We have derived a set of analytical formulae using which it is possible to determine fiber diameter (and fiber to electrode distance) from the measurement of the SFPs amplitude and negative peak duration. The analysis of the sensitivity of the determination of fiber diameter to the errors in the measurement of amplitude and negative peak duration shows that a sampling rate of 25 kHz is required so that the error in the determined fiber diameter is less than $$ 8\;\mu {\text{m}} $$. The errors in the determination of amplitude have much smaller impact on the error of the determination of $$ d $$. In order to be able to determine the fiber diameter and fiber to electrode distance, the noise level in the recorded SFP has to be low ($$ < 20$$ μV).

The ability to quantify the fiber diameter can add significantly to the SFEMG examination. It may help one to study muscle fiber diameter variability in many different muscles and thus compliment the muscle biopsy studies.

## Abbreviations

FD: Fiber density
SFEMG: Single fiber electromyography
SFP: Single fiber potential

## List of symbols

*a*: Amplitude peak-to-peak of SFP (mV)
*d*: Diameter of muscle fiber (µm)
*r*: Radial distance between muscle fiber and needle electrode (µm)
*t*_*z*_: Negative peak duration of SFP (ms)
*v*: Conduction velocity in the muscle fiber (ms^−1^)
*z*: Axial distance between muscle fiber and needle electrode (µm)
